# Hydrogenated Cs₂AgBiBr₆ double perovskites: a sustainable lead-free route toward high-efficiency solar cells

**DOI:** 10.1038/s41598-026-47055-y

**Published:** 2026-04-02

**Authors:** Ajay Kumar, Tannu, Himang Bhatia, Neha Gupta, Aditya Jain, Kaushal Kumar, Amit Kumar Goyal

**Affiliations:** 1 ECE Department, Jaypee Institute of Information Technology, Noida, 201309 India; 2 Applied Science Department, Greater Noida Institute of Technology (Engg. Institute ), Greater Noida, Uttar Pradesh 201306 India; 3 Department of ECE, Symbiosis Institute of Technology, Symbiosis International (Deemed University), Pune, 412115 Maharashtra India; 4 ECE Department, Graphic Era Deemed to be University, Dehradun, Uttarakhand India; 5 Manipal Institute of Technology, Manipal Academy of Higher Education, Manipal, 576104 India

**Keywords:** Double perovskite, Electron transport layer, Hole transport layer, Photovoltaic, Power conversion efficiency, SCAPS 1D, Solar cell, Chemistry, Energy science and technology, Engineering, Materials science

## Abstract

Lead-based perovskite absorbers have an outstanding photovoltaic efficiency, yet be hazardous. Major concerns relate to the environmental safety and the durability of the devices in the long run. As an Outcome, a Cs_2_AgBiBr_6_-based double-perovskite material has emerged as a viable lead-free solution due to its chemical stability and reduced toxicity. Although they are not yet as efficient as traditional Pb-based perovskites, recent research, particularly studies on hydrogenation-induced bandgap tuning, indicates that they can be improved. In this study, we use the computer model SCAPS-1D to critically assess the performance of a hydrogenated Cs_2_AgBiBr_6_-absorber electrode supported in an inverted p-i-n model. A parameter sweep was performed in detail to investigate the influence of thickness and defect absorber density, concentration of doping of the hole transport layer. We also assessed the performance of popular electron transport layers, which are SnO_2_ and ZnO, to detect the best charge-extracting material to use in the proposed design. The optimized device configuration, using ZnO ETL, 0.4 μm absorber thickness, and better parameters of the HTL, provides a current density (short-circuited) of 20.87 mA/cm^2^, voltage(open-circuited) of 1.4523 V, fill factor of 85.50% and 25.92% total power conversion efficiency, respectively. These results demonstrate that optimizing the parameters leads to an increase in photovoltaic efficiency of lead-free double-perovskite solar cells. This paper demonstrates the potential of Cs_2_AgBiBr_6_ Absorbers as sustainable materials in future photovoltaic innovations.

## Introduction

**Fig. 1:**
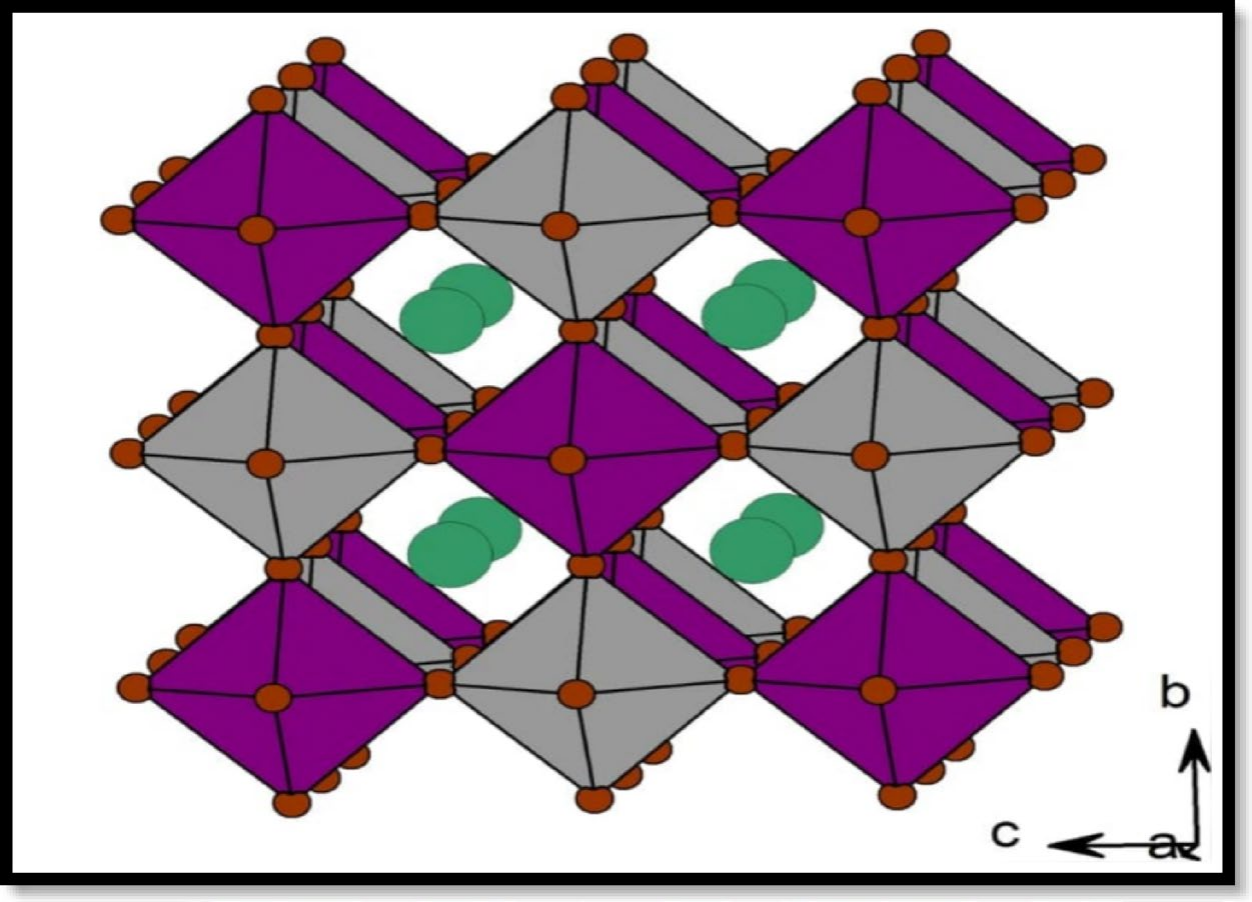
Crystal of double perovskites at a unit cell with an octahedron of corner-sharing structure. It is made of a series of $\:\left[AgB{r}_{6}{]}^{5-}\right.$(grey) and $\:\left[BiB{r}_{6}{]}^{3-}\right.$(purple) coupled octahedra of the kind utilized with Cs^+^ cations (green) occupying the A-site crystallography coordinates^21^.

Perovskite solar cells (PSCs) have quickly become a significant competitor in third-generation photovoltaic technologies. Their outstanding optoelectronic properties, including high absorption in the entire visible range, long carrier diffusion lengths, low exciton-binding energy, and defect tolerance^1–4^, have realized certified power conversion efficiencies (PCEs) exceeding 25%^5–8^. However, the reality of hazardous lead (Pb) in the B-point structure (based on the ABX_3_ format involving cations (organic/inorganic (A), a divalent (B), and one or more halides ions X) crucial to the achievement of high edge obstructs their enormous eventuality, consequently, such aspects as current voltage hysteresis, restricted stability, and lead toxin impede the implementation of such lead- containing PSCs^9^. A possible way to remove the toxic lead in the absorber is to replace Pb^2+^ with a pair of monovalent and trivalent cations, most notably Ag^+^ and Bi^3+^ to give a structure of a double perovskite with the general formula A_2_BB’X_6_ as shown in Fig. 1. The chemical Cs_2_AgBiBr_6_^10^ has attracted specific attention because it is stable, has a non-toxic composition, and has a good electronic configuration. Despite a comparatively large bandgap (2.0 Ev to 2.2 eV) of pristine Cs_2_AgBiBr_6_, recent efforts by Zhang et al. have shown that the bandgap can be efficiently hydrogenated to around 1.64 eV to provide enhanced absorption and boost device efficiency by a factor of 6.37%^11^(compared to 4.23%^13–16^. In SCAPS-1D, the hydrogenation of Cs₂AgBiBr₆ cannot be introduced explicitly at the atomic level. Therefore, the effect of hydrogenation was incorporated indirectly through modification of the absorber material parameters based on literature reports. Hydrogenation has been shown to improve optoelectronic properties of Cs₂AgBiBr₆ by tuning the bandgap and reducing trap-state density through defect passivation. Accordingly, the absorber bandgap and defect density parameters were adjusted to represent hydrogenated Cs₂AgBiBr₆, while other parameters, such as carrier mobility and density of states, were adopted from previously reported experimental and simulation studies.

Such results can be used to make further improvements by taking good care of the parameters of the devices by means of careful engineering. This development inspires the current paper to discuss the performance enhancement of hydrogenated Cs_2_AgBiBr_6_ in an inverted (p-i-n) PSC architecture in the scope of thorough numerical simulations performed with the help of the SCAPS-1D software package^16^.

The systematic variation of the properties of the absorber-layer, such as thickness and defect density, and the effect of the thickness of the hole transport layer (HTL) and doping concentration, are analyzed. We also consider various electron transport layer (ETL) materials, with the main focus on comparing SnO2^17,18^ and ZnO^18–20^ to identify the ETL that is most favourable for efficient carrier extraction. With the help of this multiplex optimization strategy, we are going to find a range of structural and material parameters that will be able to considerably improve the performance of lead-free perovskite solar cells. The findings indicate the high potential of hydrogenated Cs_2_AgBiBr_6_ absorbers and offer useful information in informing future experimental endeavours on the development of environmentally friendly and high-performance photovoltaic equipment.

##  Materials and device modelling

**Fig. 2:**
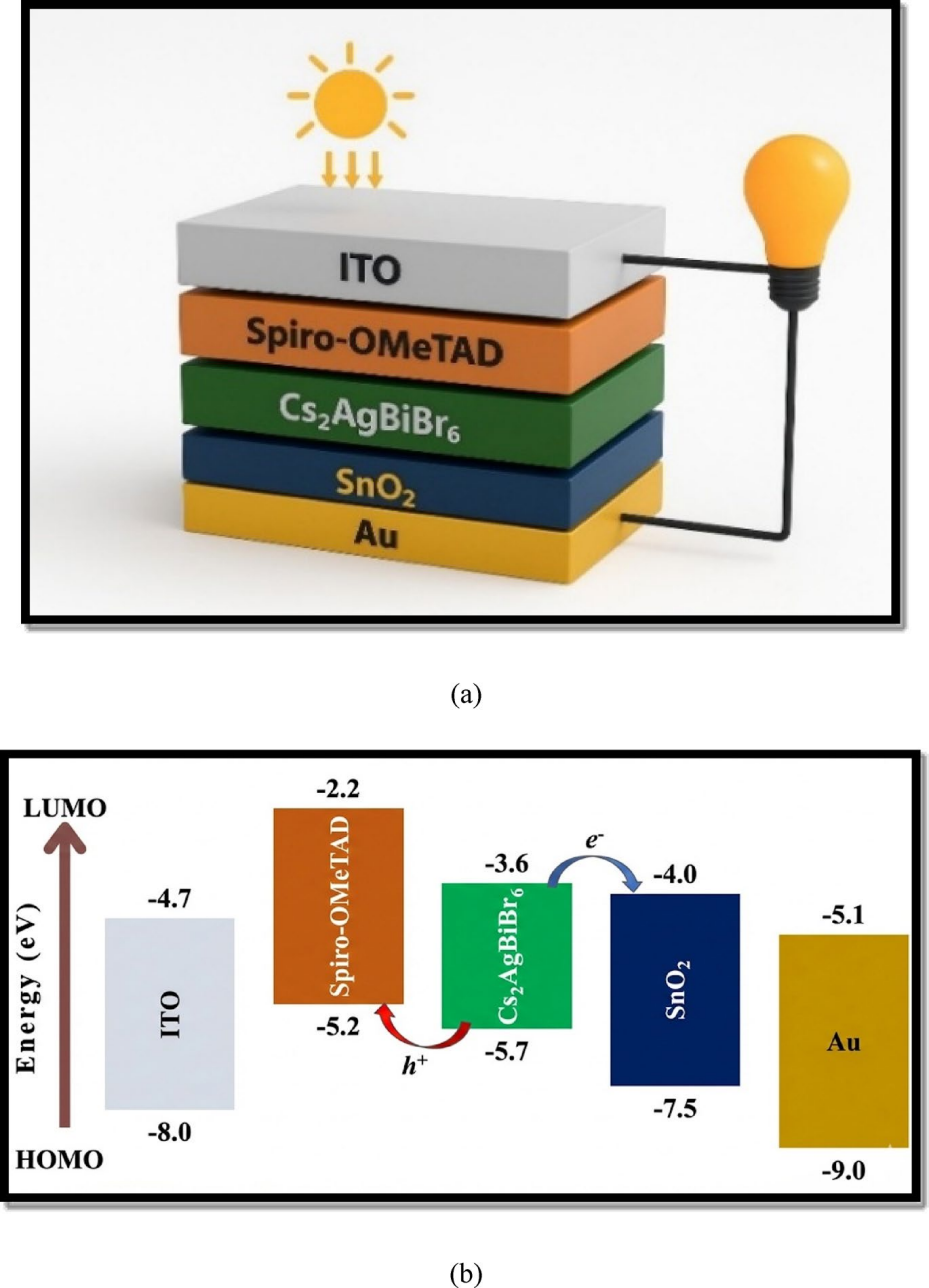
(**a**) The architectural layout of the device of Cs_2_AgBiBr_6_-based lead-free perovskite solar cells (PSCs) (**b**) Energy band diagram for each layer used in the architecture.

The paper is an expansion of the research of Zhang et al., who were conducting research on the hydrogenated Cs_2_AgBiBr_6_^11^, and they were able to make the conclusion that the narrowing of the bandgap of the Cs_2_AgBiBr_6_, allows better photovoltaic operation than the pristine silicon. Based on these observations, we used SCAPS-1D software version 3.3.12 on which we modelled and eventually optimized the non-toxic double-perovskite solar cell with the hydrogenated absorber material. SCAPS-1D is an extremely popular one-dimensional semiconductor simulation software created at the University of Gent to analyse multilayer photovoltaic structures in steady-state. The following design of the LFDP solar cell based on a standard of 300 K temperature, 1000 W/m^2^ irradiation intensity and air mass AM 1.5 G, included the back contact of the gold and indium tin oxide commonly referred to as ITO as the frontal contact, Spiro-OMeTAD^22^ as HTL and SnO_2_ as ETL as illustrated in Fig. 2(a) and the energy band diagram for each layer used are shown in the Fig. 2(b). This arrangement employs a reversed (p-i-n) design, and, in this design, light passes through the cell on the HTL side. The electronic and optical parameters of each layer were chosen according to the previous experimental and theoretical investigations of the perovskite and double-perovskite materials. A table (Table 1)^23–28^ has been prepared to indicate the main material constants, which were directly entered as simulation input, and they were fixed unless optimization studies.

Moreover, Table 2^23–28^ provides the distribution of the defects in every layer. They were assumed to be neutral traps, the cross-sections of which were taken to be 1 × 10^− 15^ cm^− 2^, both for the electron and the hole. The absorber.

defects represented a Gaussian energetic distribution, and both transport layers had single-level traps. A structural parameter-engineering strategy was taken with the goal of identifying parameters that optimize cell conversion efficiency. The variables that were altered in a sequential manner were as follows: (a) Electron Transport Layer (ETL) Material, SnO_2_ (baseline) and ZnO (alternative with higher mobility and favourable band alignment), (b) Absorber Layer Properties such as Thickness: varied between 0.2 and 1.2 μm, Defect density (N_t_): varied between 10^12^ and 10^18^ cm^− 3^.(c) Hole Transport Layer (HTL) Properties such as Thickness: ranged between 0.01 μm and 0.1 μm and Acceptor doping concentration (N_A_): ranged between 1 × 10^14^ and 1 × 10^19^ cm^− 3^. Key photovoltaic parameters that were measured during each simulation included: Open-circuit voltage (V_oc_), short-circuit current density (J_sc_), fill factor (FF), and power conversion efficiency. This sequential optimization allows seeing the effect of each parameter on the performance of the devices clearly and determines the synergistic effect among layers. SCAPS takes one approach towards the solution to these algorithms, the Poisson equation as seen in Eq. (1). The equations of continuity of electrons and holes, mentioned in Eq. (2) and Eq. (3) respectively^11^.

1 $$\:\frac{d}{dx}\:\left(-\epsilon\:\left(x\right)\frac{\:d\psi\:}{dx}\:\right)=q\left[p\left(x\right)-n\left(x\right)+{N}_{D}^{+}\left(x\right)-{N}_{A}^{-}\left(x\right)+{\:p}_{t}\left(x\right)-{n}_{t}\left(x\right)\right]$$

2 $$\:\frac{{d}_{Pn}}{dt}={G}_{p}-\frac{{p}_{n}-{p}_{{n}_{0}}}{{\tau\:}_{p}}+{p}_{n}{u}_{p}\frac{d\epsilon\:}{dx}+{\mu\:}_{p}\epsilon\:\frac{d{p}_{n}}{dx}+{D}_{P}\frac{{d}^{2}{p}_{n}}{d{x}^{2}}$$

3 $$\:\frac{d{n}_{P}}{dt}={G}_{n}-\frac{{n}_{p}-{n}_{{p}_{0}}}{{\tau\:}_{n}}+{n}_{n}{\mu\:}_{n}\frac{d\epsilon\:}{dx}+{\mu\:}_{n}\epsilon\:\frac{d{P}_{P}}{dx}+Dn\frac{{d}^{2}{n}_{p}}{d{x}^{2}}$$

**Table 1:** Electrical and optical properties of materials used in simulation.

Parameters	Spiro-OMeTAD (HTL)	Cs2AgBiBr6 (Absorber)	SnO_2_ (ETL)	ZnO (ETL)
Thickness(µm)	0.06	0.14	0.05	0.05
Band gap (eV)	2.9	1.61	3.6	3.3
Affinity of electrons χ *(*eV)	2.2	3.72	4.5	3.7
Permittivity (Dielectric)	3	5.8	8	9
CB efficient state Density (cm^− 3^)	2.5 × 10^19^	2 × 10^18^	2.2 × 10^18^	2.2 × 10^18^
VB efficient state Density (cm^− 3^)	1.8 × 10^19^	1 × 10^18^	1.8 × 10^18^	1.8 × 10^19^
Mobility of electrons (cm^2^/V. s)	2 × 10^− 4^	9.28	15	100
Mobility of holes (cm^2^/V. s)	2 × 10^− 4^	9.28	15	25
Concentration of the acceptor dopant (cm^− 3^)	1 × 10^17^	0	1 × 10^18^	1 × 10^18^
Concentration of the donor dopant (cm^− 3^)	1 × 10^15^	1 × 10^15^	0	0

**Table 2:** Density values of defects within cell layers.

Parameters	ETL	HTL	Absorber
Type of Defects	Neutral	Neutral	Neutral
Cross-sectional capture for electrons σ_n_ (cm^− 2^)	1 × 10^− 15^	1 × 10^− 15^	1 × 10^− 15^
Cross-sectional capture for holes σ_p_ (cm^− 2^)	1 × 10^− 15^	1 × 10^− 15^	1 × 10^− 15^
Distribution of Energies	Single	Single	Gaussian
Level of energy with respect to Ev (above Ev) (eV)	0.6	0.650	0.6
Characteristic energy(eV)	0.1	0.1	0.1
Overall Density N_t_ (cm^− 3^)	1 × 10^15^	1 × 10^15^	1 × 10^16^

## Results and discussion

It presents a detailed analysis of the results of the numerical simulations and highlights the effects of important material and structural parameters on the photovoltaic efficiency of the proposed low-bandgap double perovskite (LFDP)-based perovskite solar cells (PSCs). The optimization scheme of the work follows a systematic and stepwise approach, which starts with the identification and optimization of the transportation layer of electrons, then the properties of the active layer, such as its thickness and density of defects, and finally optimization of the transportation layer of holes, its thickness and its doping concentration. Using this combined parameter engineering, an effective strategy of improvement of device characteristics, such as open-circuited voltage, short-circuited density of current, fill factor, and power conversion efficiency, is developed. This approach further reveals the interplay of optical and electronic processes, which eventually determine the PCE of the cell.

### Impact of the transportation layer of electrons on LFDP cell performance

**Fig. 3:**
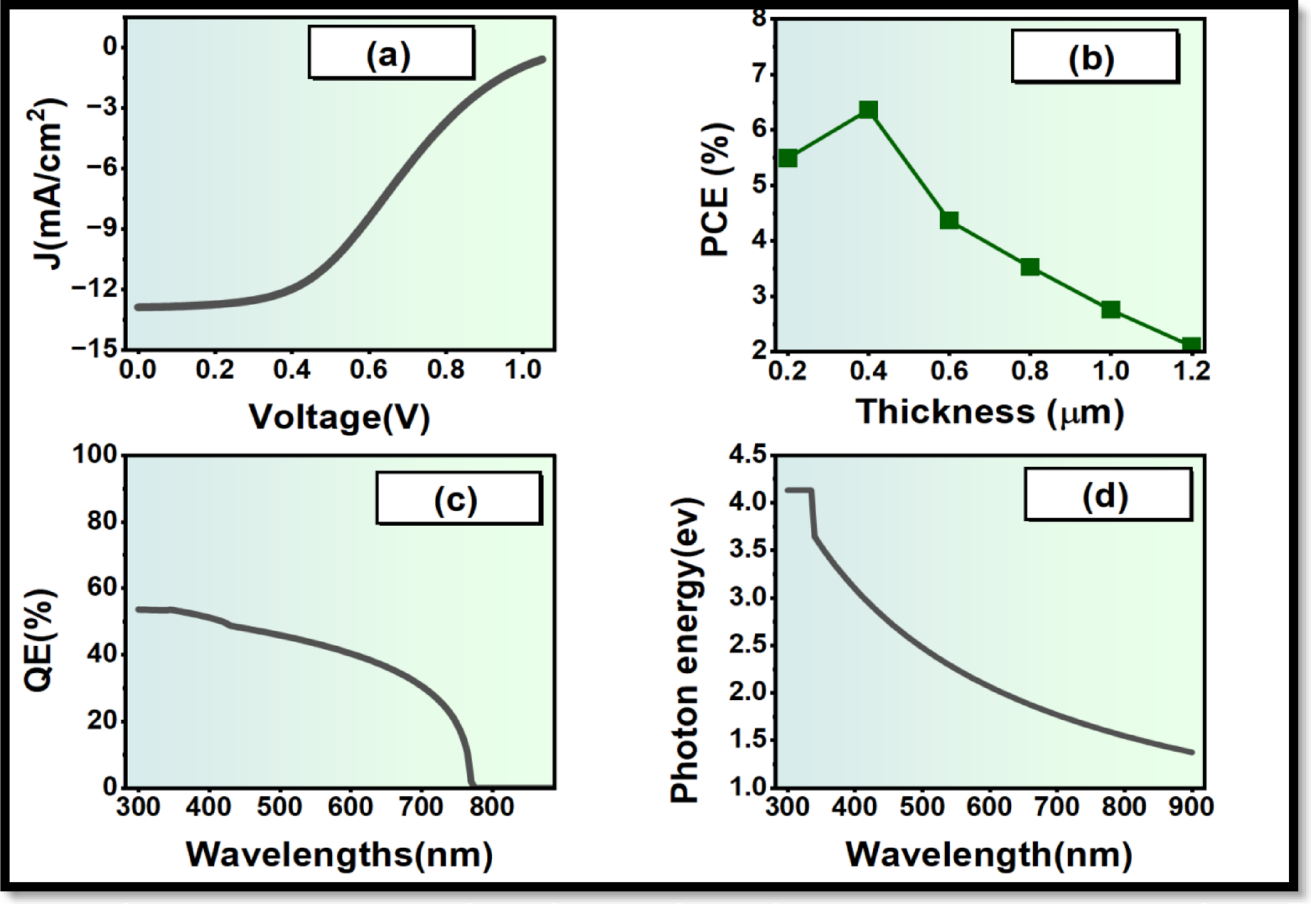
Photovoltaic characteristics of the SnO_2_-based perovskite solar cell: (**a**) J-V characteristics when illuminated, (**b**) influences of the absorber thickness on photovoltaic characteristics, (**c**) external quantum efficiency (QE) versus wavelength, (**d**) photon energy versus wavelength.

**Fig. 4:**
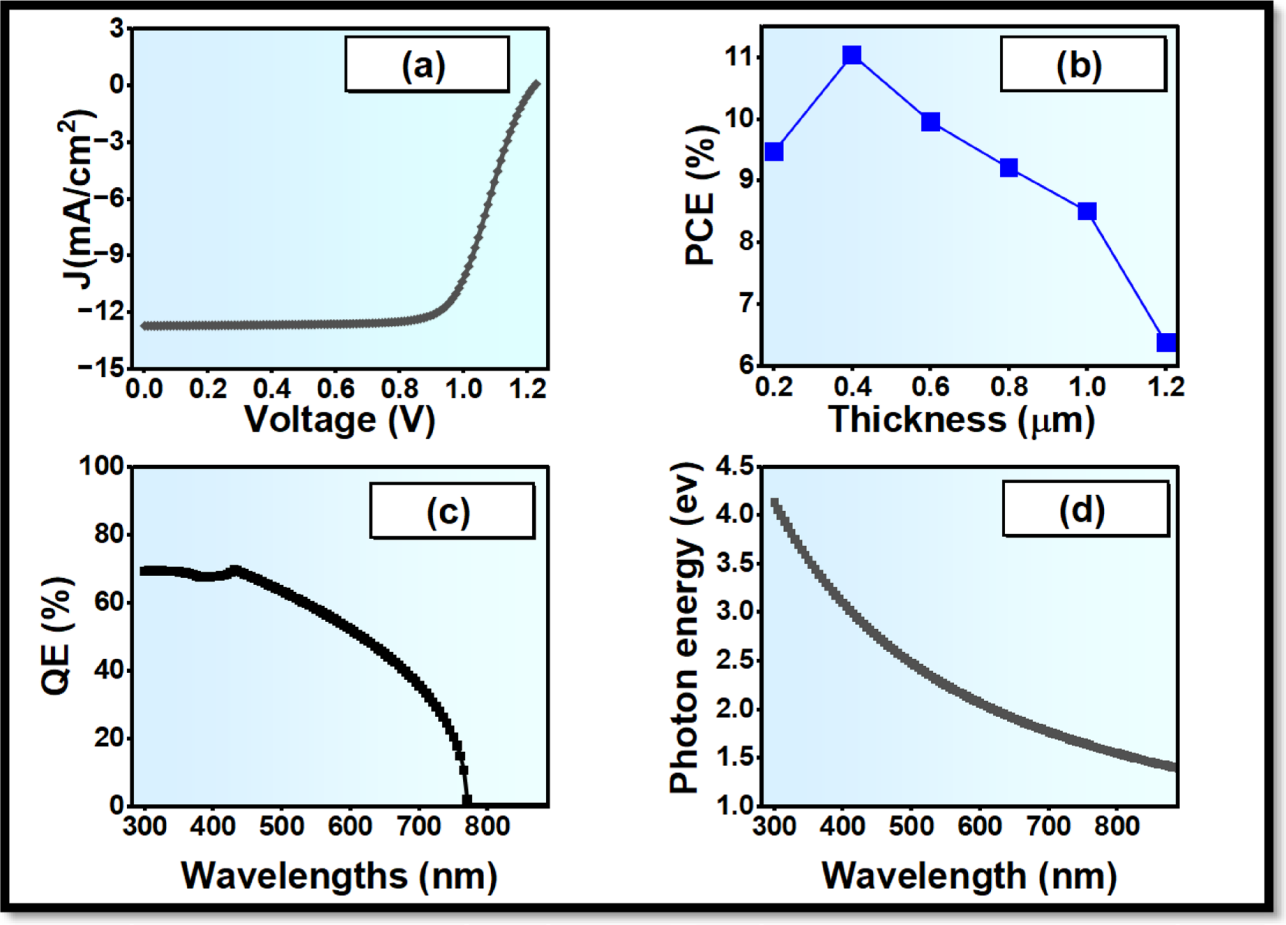
Photovoltaic characteristics of the ZnO-based perovskite solar cell: (**a**) J-V characteristics when illuminated, (**b**) influences of the absorber thickness on photovoltaic characteristics, (**c**) external quantum efficiency (QE) versus wavelength, (**d**) photon energy versus wavelength.

The Electron transportation plays a significant role in offering the extraction of the electrons of the absorber as well as lowering the recombination across the interface. The two ETL materials, SnO_2_ and ZnO, were also considered, as they are also commonly utilized among the inverted perovskite designs, and the two have various electronic properties. SnO_2_ was taken as the reference ETL due to the fact that it possessed a good degree of transparency, chemical stability and also due to the fact that it was known to be compatible with perovskite absorbers. However, ZnO possesses favourable characteristics that can be further enhanced to enhance charge transport, and they are: Higher electron mobility, compared transparency, more favourable conduction-band alignment with Cs_2_AgBiBr_6_, and Lower interface recombination potential. Figures 3 and 4 depict the photovoltaic performance of PSCs having SnO_2_ as ETL and then ZnO, respectively. This absorption window (300–800 nm) is virtually identical and indicates that the improvement of the performance is caused not by optical changes but by the enhancement of charge extraction. The cell containing SnO_2_ has a PCE of about 6.38% as well as a replacement with ZnO elevates the cell performance to 11.03%, as depicted in Fig. 3(b) and Fig. 4(b). Efficiency has been greatly enhanced, which proves the value of enhanced band alignment and low carrier losses in the ZnO-based designs. Spectra for the quantum efficiency of the two configurations show variations differently. The best QE values of the devices based on SnO_2_ reach approximately 55% in view of those based on ZnO, with a peak of around 70% as shown in Fig. 3(c) and Fig. 4(c). Consequently, ZnO is selected as the ETL that is to be utilized in the new optimization studies.

###  Effect of the absorber (LFDP) thickness on solar cell performance

An essential component of a photovoltaic system is the absorber layer, which determines the solar radiation that can be transformed into useful electrical energy. In the systems of using the double perovskite or lead-free perovskite, the changes in structural, optical, and electronic properties affect the performance to a significant extent. The rates of photogeneration, the lifetime of charge carriers, the probability of recombination, and the dynamics of transport depend heavily on two important factors, i.e., the absorber thickness and the defect density (N_t_). It is important to optimize these parameters in order to come up with efficient and affordable solar cells. An efficient absorber layer is well-designed and also enhances absorption, reduces non-radiative recombination, and helps in efficient extraction of charge. In this experiment, we systematically changed the absorber thickness and defect density to determine the best operating conditions in LFDP-based perovskite solar cells.

**Fig. 5:**
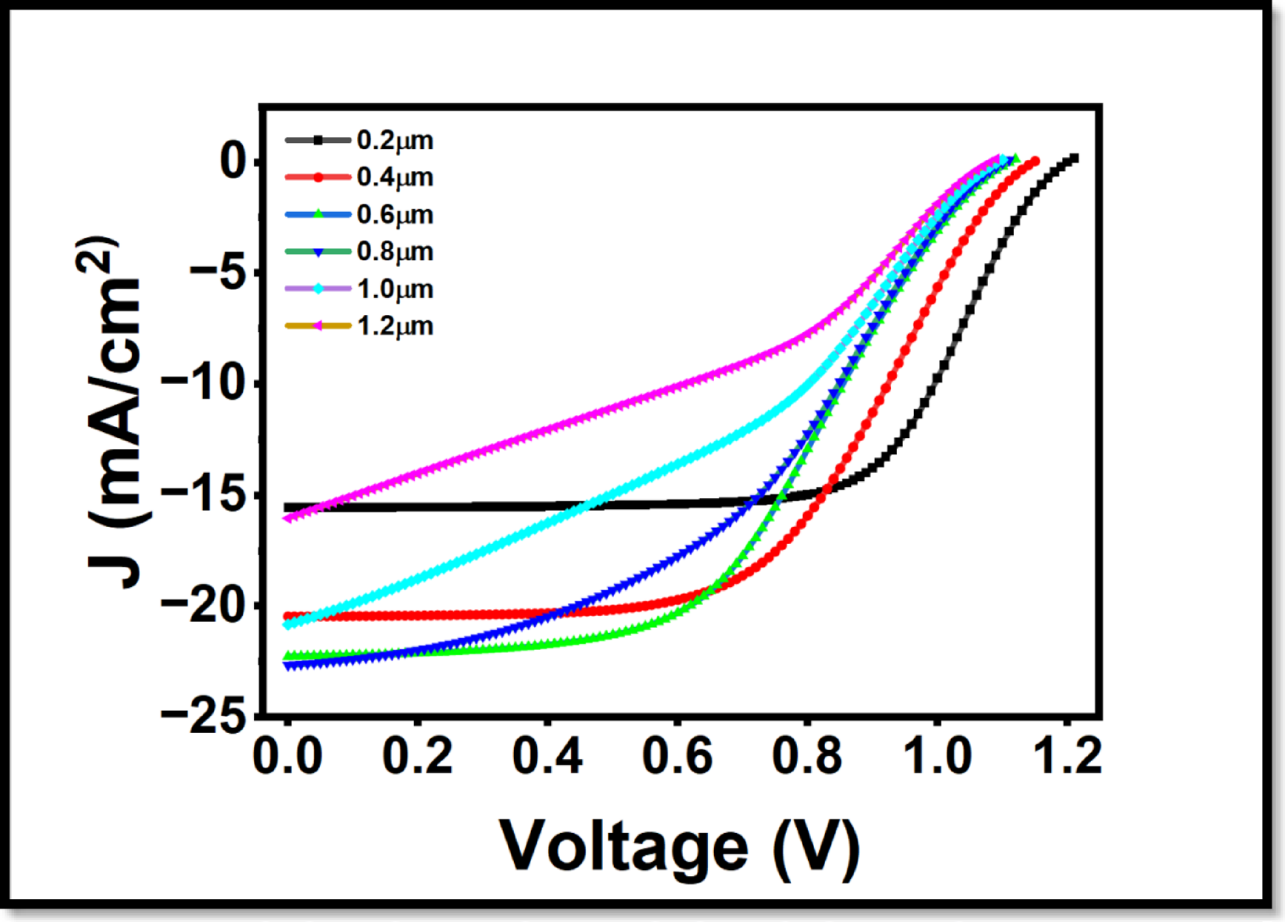
Influence of absorber layer thickness (0.2–1.2 μm) on current density voltage (J-V) characteristics of the perovskite solar cell under irradiation.

The depth of the layer of the absorber has a strong effect on light-harvesting efficiency and charge carrier flux. The thinner absorber can possibly not absorb a significant amount of incoming photons and cause less photogeneration and a following decrease in Jsc. Over dense layers, on the contrary, increase the likelihood of recombination because of long distances of transportation and short diffusion carriers. The range of thickness that was studied was between 0.2 μm and 1.2 μm in order to explore the thickness effect. The performance trends are witnessed in terms of the results. Figure 5 indicates the thickness of the absorber and its effects on the J-V characteristics.

**Fig. 6:**
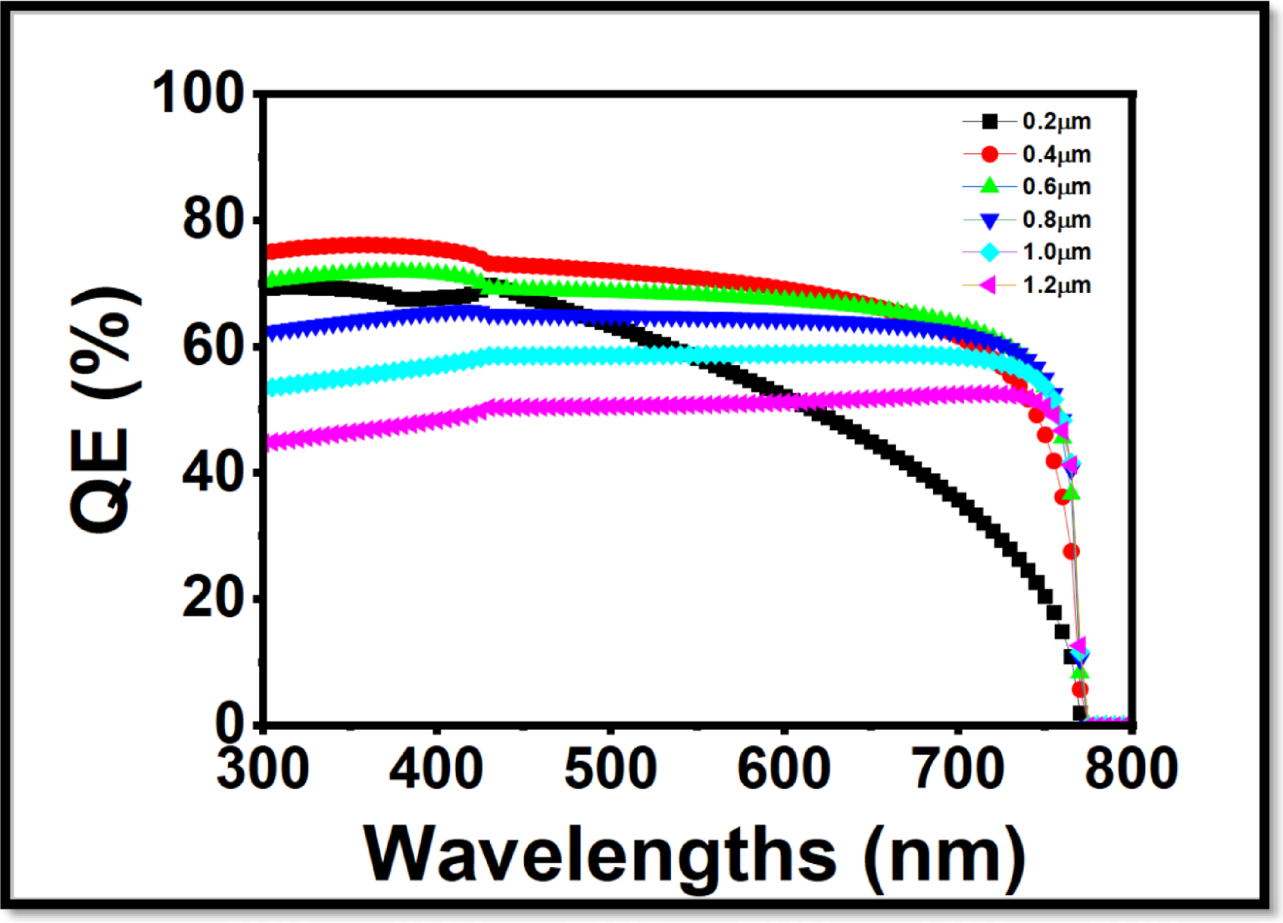
Effects of various absorber thicknesses on Quantum efficiency (QE) of the perovskite solar cell.

Further, Fig. 6 shows that the QE response displays a dual dependency on absorber thickness. At shorter wavelengths **<** 450, a decrease in QE is observed with increasing thickness. This is attributed to the fact that high-energy photons are absorbed near the illuminated surface; in thicker devices, the resulting charge carriers must traverse a longer path through the bulk to reach the electrodes, increasing recombination probability. However, at extended wavelengths > 600 nm, thicker layers (up to 0.4 μm) are used to enhance the likelihood of light absorption, as the increased volume allows for the capture of photons with lower absorption coefficients.

**Fig. 7:**
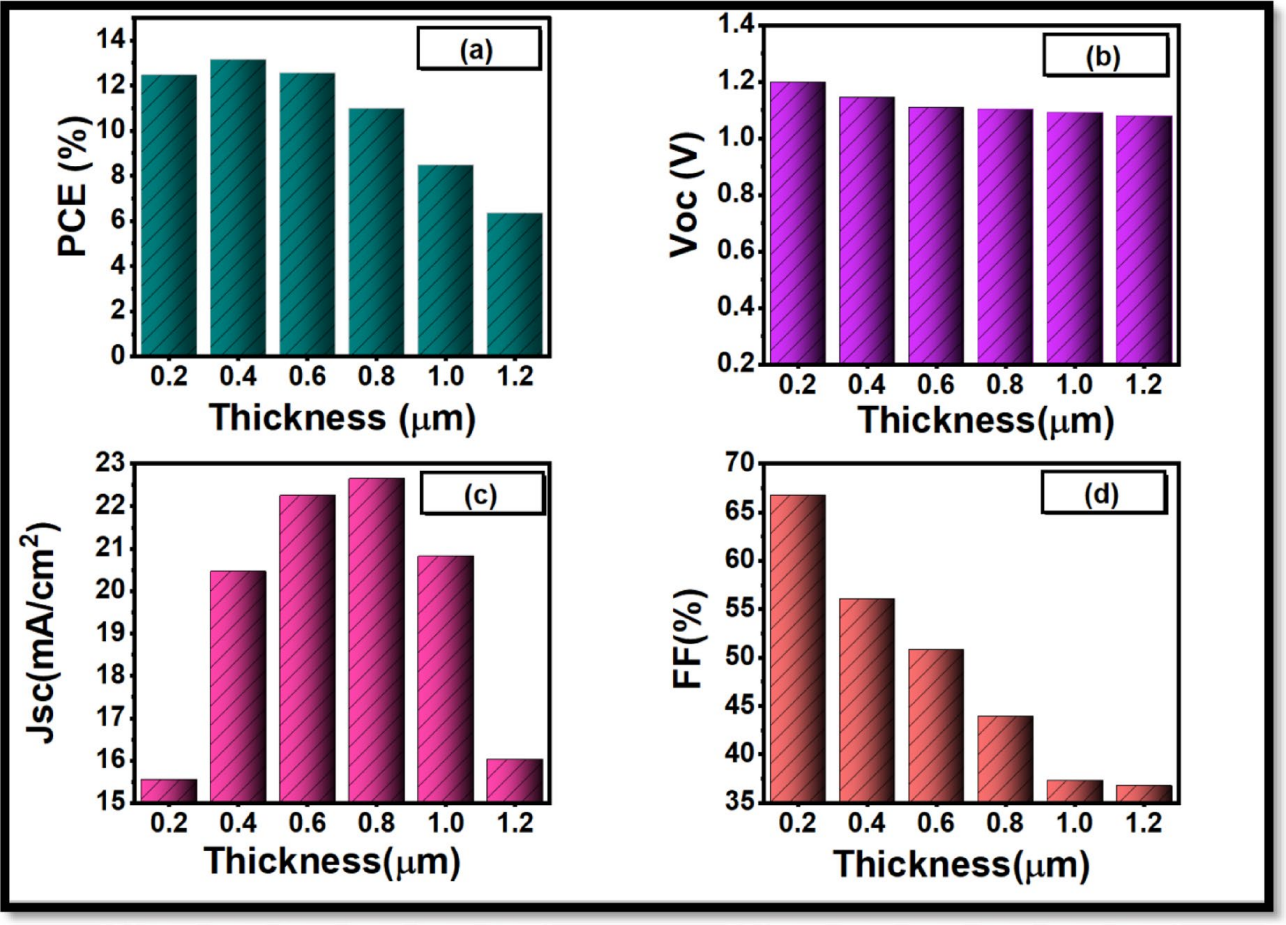
Dependence of important electrical parameters on absorber layer thickness: (**a**) power conversion efficiency (PCE), (**b**) open-circuit voltage V_oc_ (**c**) short-circuit current density J_sc_, and (**d**) fill factor (FF).

Figure 7(a) depicts the PCE curve, indicating that efficiency starts increasing with a rise in the absorber thickness; this is because more photons are absorbed, and a larger number of electrons and holes can form a pair in the otherwise thick layers. Consequently, the power conversion efficiency (PCE) rises and peaks at 0.4 μm with the value of 13.2%. By doing so, we may pass a conclusion that the thickness of the absorber is 0.4 μm; further than this, the PCE reaches a minimum, and this also demonstrates the considerable contribution of the absorber thickness in the operation of the device. The open-circuit voltage (V_oc_), however, is rather independent of the thickness range as shown in Fig. 7(b); it is expected to be so since V_oc_ only varies primarily with the energetic alignment and recombination events but not with the geometry of the absorber. There is also the shortcircuit current density (J_sc_) that is also found to have significant increase of 15.29 to 23.31 mA/cm^2^ as demonstrated in Fig. 7(c) with increase in thickness between 0.2 μm and 0.8 μm but not very easily, it also begins to decrease with an increase in thickness to a value of 15.9 mA / cm^2^ at 1.2 μm. Fill factor shows a decline when thickness increases, as depicted in Fig. 7(d). In conclusion, light absorption, recombination rates and photon energy at different wavelengths and thicknesses show both complex interdependence in determining quantum efficiency properties of LFDP materials. With this tremendous finding as a basis, 0.4 μm has been chosen as a thickness in the given study.

### Impact of defect density N_t_ of the absorber layer

The absorber thickness is vital in estimating the optical absorption flair of the absorber, yet the density of the defects has a huge impact in caliber of the electronic properties of the substance. Non-radiative recombination centers are like various defects, such as vacancies, grain boundaries, dislocations, and deep trap state which decrease carrier lifetime and each of which decreases photocurrent to a great extent. It therefore involves ensuring there is a reduced concentration of defects so as to have as much successful charge collection and reduce as much as possible the losses that occur during recombination.

**Fig. 8:**
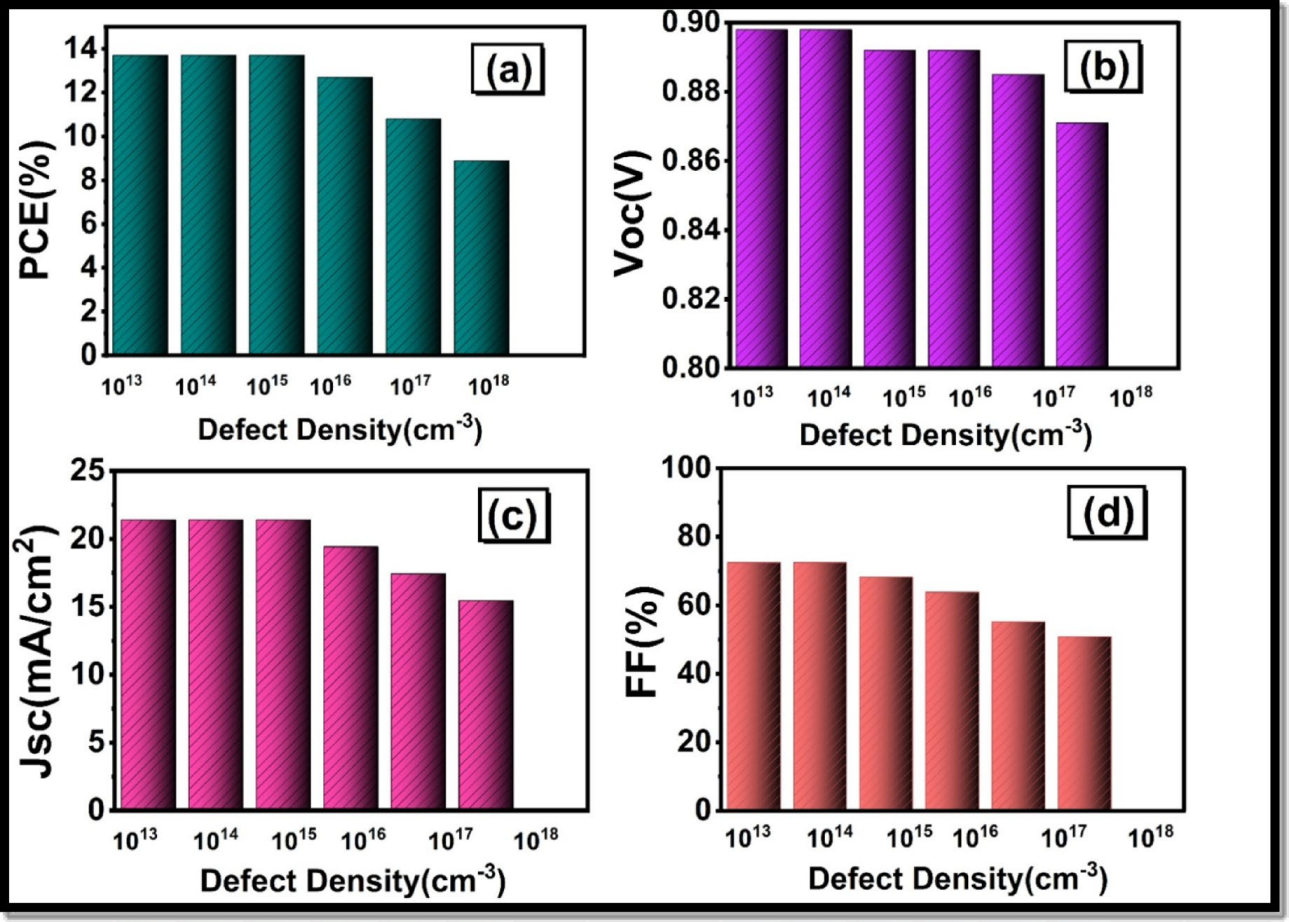
Dependence of important electrical parameters with defect density of the absorber: (**a**) power conversion efficiency (PCE), (**b**) open-circuit voltage V_oc_, (**c**) short-circuit current density J_sc_ and (**d**) fill factor (FF).

The LFDP absorber layer was a wide range, and the density of defects of the absorber layer varied in the study from 10^13^ cm^− 3^ to 10^18^ cm^− 3^. It is a wide range that allows making a complex evaluation of the influence of the intrinsic quality of the material on the work of the devices. As can be seen in Fig. 8(a), when the defect density (N_t_) is less than 10^15^ cm^− 3^, the conversion efficiency of power (PCE) only reduces marginally since quality films are exposed to minimal recombination losses. At some point, however, N_t_ exceeds the point of 10^16^ cm^− 3^ to 10^18^ cm^− 3^, there is a sharp decrease in performance as the charge carriers are heavily trapped and the non-radiative recombination occurs. At very high defect densities (over 10^18^ cm^− 3^), the PCE also causes a dramatic reduction in the device hence inefficient. Using the PCE graph, it is possible to conclude that the optimal density of defects is 10^14^ cm^− 3^. Open-circuit voltage (V_oc_) was virtually unchanged initially and then started to decrease as defect increases as shown in Fig. 8(b).

The fill factor (FF) drops to approx. 50% of the former 73.2% as shown in Fig. 8(d), leading to a radical drop in the photovoltaic effectiveness and the density of the short-circuit current (J_sc_) stays constant until a defect density of 10^15^ cm^− 3^, at which the short-circuit current density drops significantly, to 16 mA/cm^2^ as depicted in Fig. 8(c).

### Influence of the HTL thickness on LFDP solar cell performance

The extraction of the holes layer is also quite a proper constituent of the inverted perovskite solar cells (p-i-n) structures design that quantifies the manner in which holes are extracted, charge interfaces recombination, but also how the overall device is rendered stable. The holes in the HTL are to be extremely mobile; the holes should be in good energetic alignment with the absorber and the transparency to optimally transmit the light to the layer. Spiro-OMeTAD is one of the most popular materials that has been utilized in a wide range as HTL, and it has a high level of transparency (bandgap 2.9 eV), processability, and the level of energy. It possesses huge transmission in the visible spectrum that lowers parasitic absorption losses and lets more photons reach the LFDP absorber. It is also favorably positioned with the highest occupied molecular orbital (HOMO) position, which makes the extraction of holes easy. However, despite these positive features, Spiro-OMeTAD-based PSCs activity is affected by the thickness and the doping concentration.

**Fig. 9:**
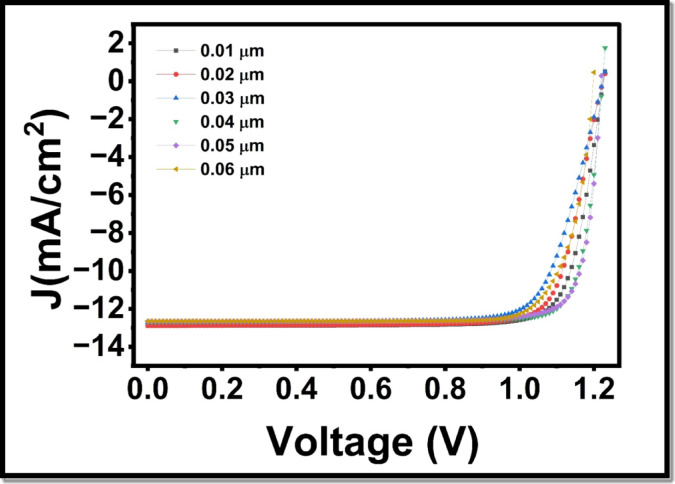
Influence of HTL layer thickness (0.01–0.6 μm) on current density voltage (J -V) characteristics of the perovskite solar cell.

To examine these effects, we adjusted these parameters and examined their effects on the attributes of the devices. As the thickness of the HTL is more than an optimal thickness, there is an increase in resistive losses that maximizes the fill factor and the total power conversion efficiency (PCE). Conversely, excessively thin layers were not capable of giving homogeneous coverage, and thus, a high recombination at the interface is possible. Similarly, doping also modulates conductivity and charge transfer at the interfaces, but too much doping can result in morphological degradation or perversity. The sensitivity can be optimized to a large extent to enhance the stability and performance of the said devices, and hence, interfacial engineering is highly critical in the design of PSCs.

**Fig. 10:**
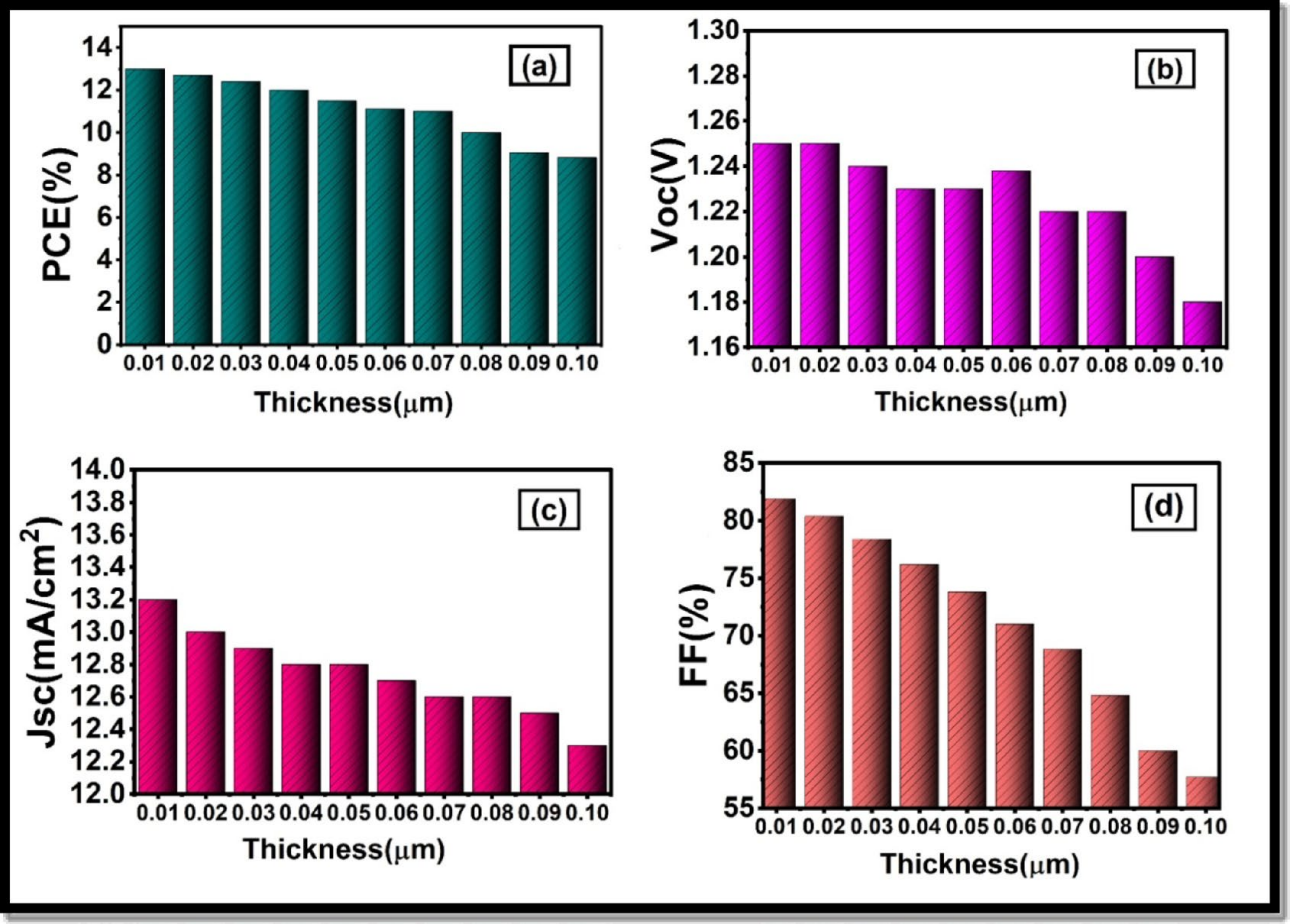
Dependence of important electrical parameters on HTL layer thickness: (**a**) power conversion efficiency (PCE), (**b**) open-circuit voltage V_oc_, (**c**) short-circuit current density J_sc_, and (**d**) fill factor (FF).

In the previous research, the importance of the thickness of the HTL on the performance of PSC has been highlighted. Precisely, a very thin HTL layer does not provide a thin enough cover to the absorber layer. On the other hand, an increased HTL layer increases the chance of recombination as the path length travelled by charge carriers is longer, and the electrical resistance is higher in the device. Thus, it is essential to control HTL thickness closely to ensure that the irregular layer of perovskite is completely covered without causing an increase in the series resistance in the devices. Consequently, we carried out research to find the best thickness of HTL. We changed the HTL thickness to 0.01 μm and 0.1 μm and evaluated the photovoltaic efficiency. Figure 9 depicts the effect of the HTL thickness on the current density voltage characteristics, and Fig. 10 illustrates its impact on various electrical characteristics such as FF (Fill factor), short-circuited current density, Jsc, open-circuit voltage Voc and PCE. The PCE curve further demonstrates that the increase in the thickness of the HTL results in a reduction in the PCE, as shown in Fig. 10(a), primarily due to the fact that the thinner the layer, the more light is transparent and therefore the more light can be absorbed by the LFDP layer. This leads to the production of more carriers, thus resulting in an increase in FF, a crucial measure of the effectiveness of the device in converting the incoming light into electric current. V_oc_ remains constant with different thicknesses as shown in Fig. 10(b), whereas Jsc decreases between 12.02 mA/cm^2^ and 1 mA/cm^2^, as depicted in Fig. 10(c), which is in the thickness range 0.01 μm to 0.1 μm. There is also a great enhancement in FF as shown in Fig. 10(d), where the FF goes up to 24 at a rate of 19.02.

### Impact of the HTL doping concentration N_A_

**Fig. 11:**
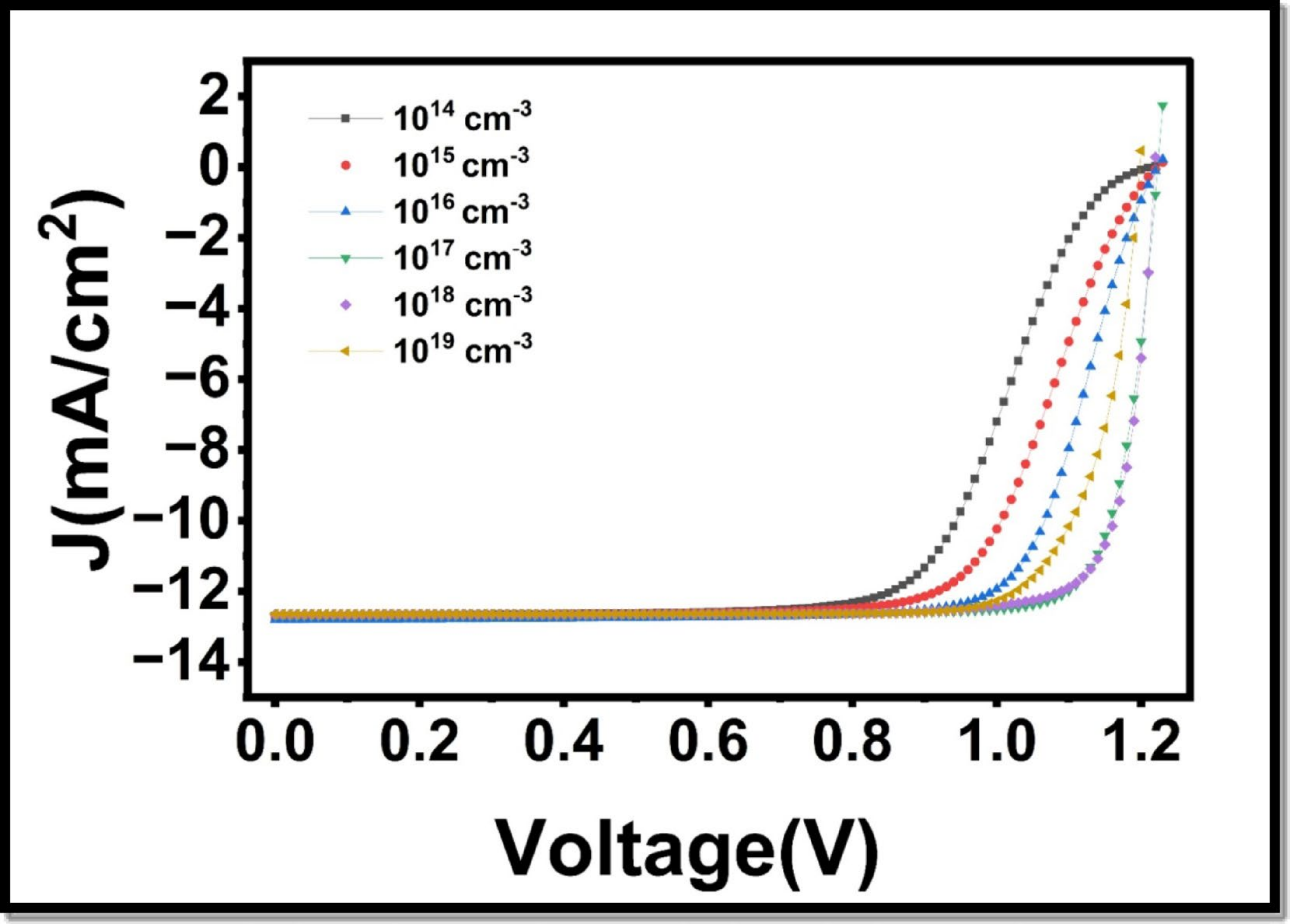
Influence of HTL doping concentration (N_A_) on current density voltage (J-V) characteristics of the perovskite solar cell.

**Fig. 12:**
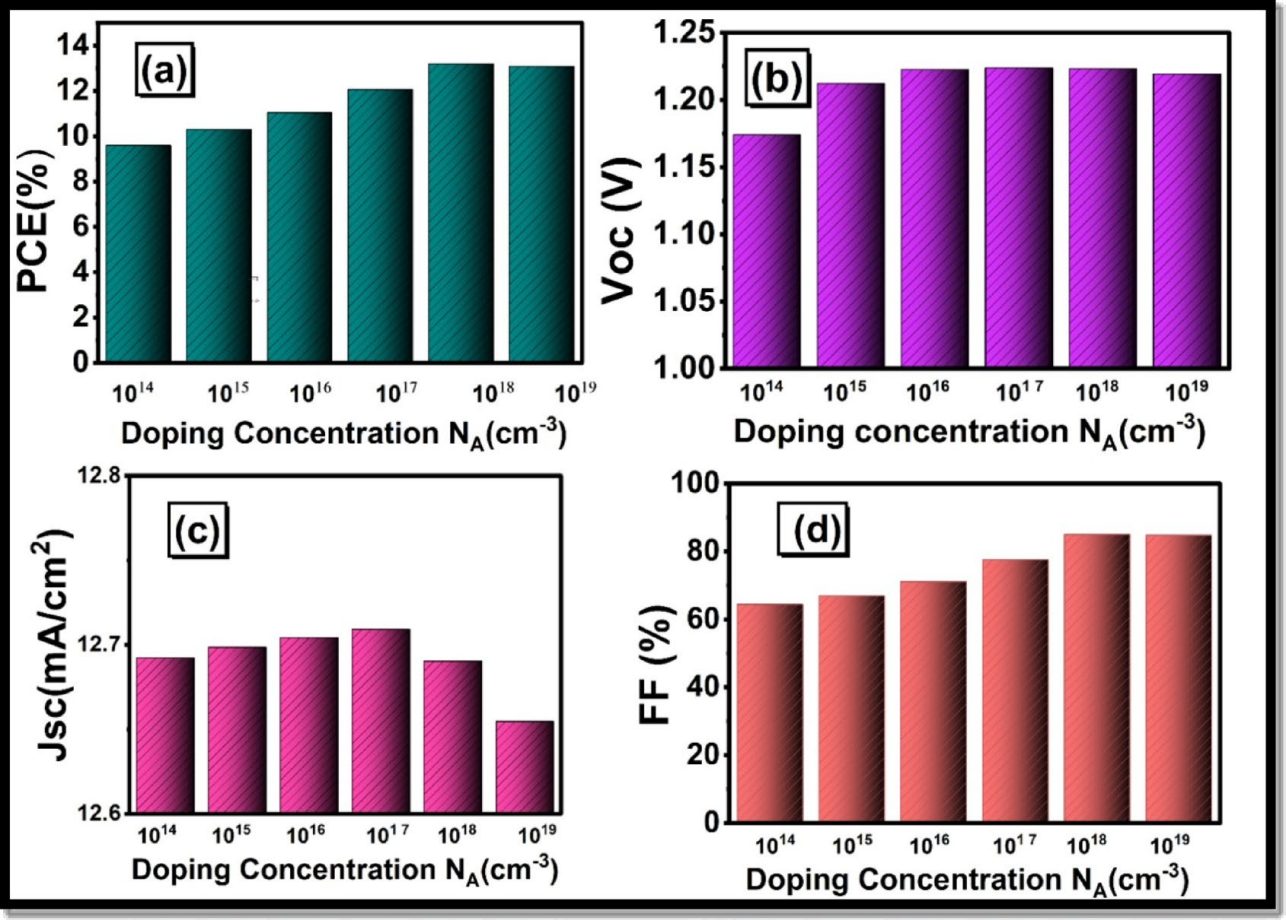
Dependence of important electrical parameters on HTL Doping Concentration (N_A_): (**a**) power conversion efficiency (PCE), (**b**) open-circuit voltage V_oc_ (**c**) short-circuit current density J_sc_, and (**d**) fill factor (FF).

To increase the performance of HTLs, one must not only focus on the thickness of the layer, but also the N_A_ concentration and its effect on the photovoltaic characteristics of PCE, FF, V_oc_, and J_sc_ in PSCs. In this experimental study, the first doping concentration N_A_ = 1 × 10^15^ cm^− 3^ was used. In our numerical study, we studied the influence of changing N_A,_ which is 1 × 10^14^ to 1 × 10^18^ cm^− 3^ and kept the thickness constant at the initial value of 60 nm. As shown in Fig. 11, the effect of the HTL doping on J-V characteristics of the PSCs can be seen. Figure 12(a) and Fig. 12(d) revealed that there was a significant growth in photovoltaic efficiency and an enhancement in FF. At a doping concentration of about 10^18^ cm^− 3^ or 10^19^ cm^− 3^, the highest PCE attains 13.3%, and the FF increases to 85.04. It is important to note that the fill factor and PCE increase at increased levels of doping, unlike the situation with HTL thickness. Figure 12(b) illustrates that Voc remains almost constant and shows a negligible change on increasing the doping concentration. In particular, both J_sc_ exhibit a slight decrease with the increase in doping concentration, that is, after 10^17^ cm^− 3^ at the same thickness of the HTL as reflected in Fig. 12(c). One can attribute this improvement in performance to HTL doping, which elevates the movement of charge carriers, particularly holes. Increased carrier mobility facilitates free movement of charges within the material, thereby reducing resistive losses and increasing the overall conductivity of the device. Doping decreases the recombination by introducing additional charge carriers to overcome the traps and defects within the substance. Moreover, doping keeps the energy levels in proper alignment, hence enhancing the efficiency in the charge transfer.

**Fig. 13:**
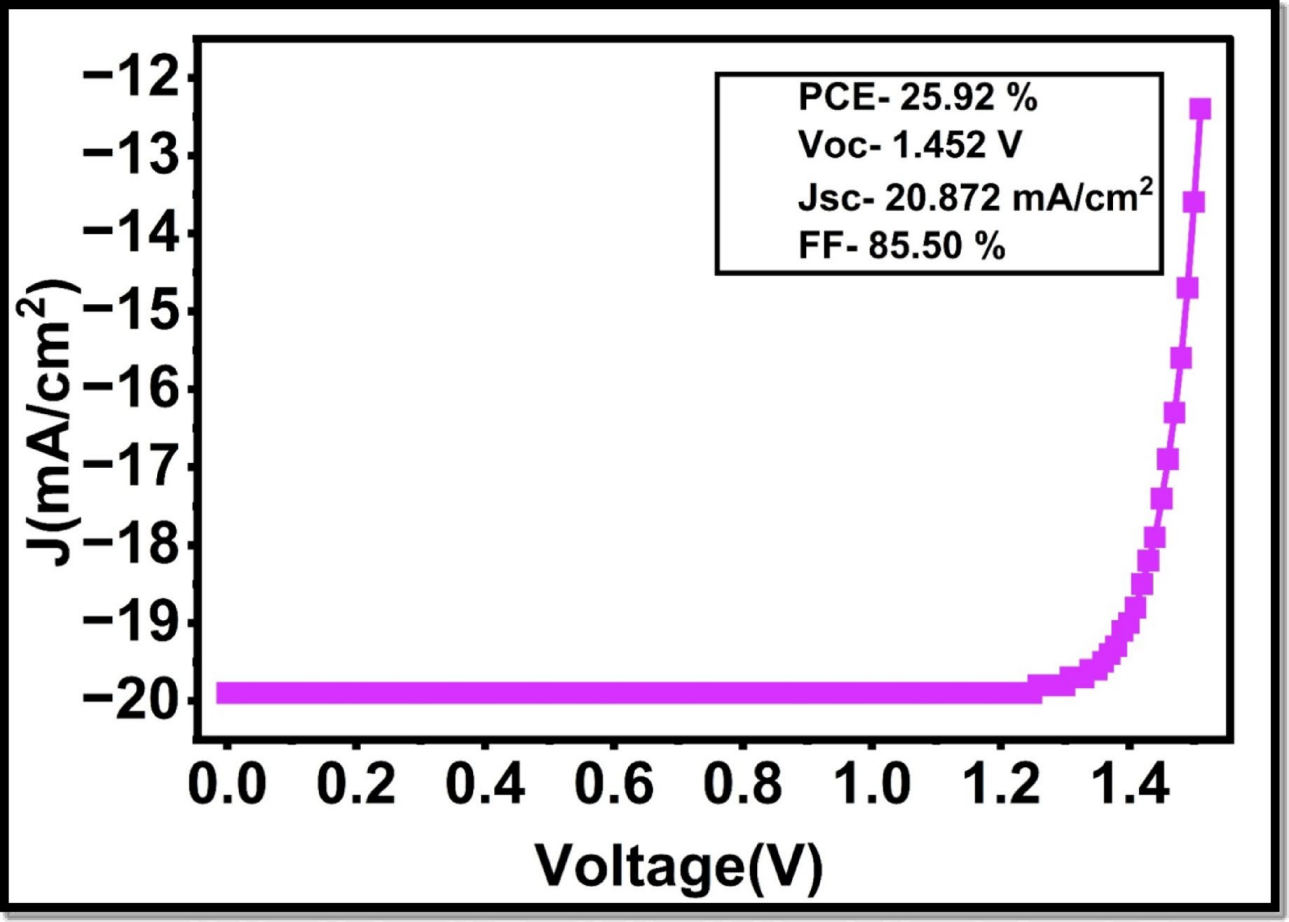
Final J-V characteristics of optimized double Perovskite solar cell (Cs_2_AgBiBr_6_) with figure of merits.

Figure 13 shows the final J-V characteristics of optimized double perovskite solar cell (Cs2AgBiBr6) with the figure of merits. The optimized device configuration, using ZnO ETL, 0.4 μm absorber thickness, and better parameters of the HTL, provides a current density (short-circuited) of 20.87 mA/cm^2^, voltage(open-circuited) of 1.4523 V, fill factor of 85.50% and 25.92% total power conversion efficiency, respectively.

##  Conclusion

Many years later, the efficacy of lead-free Cs_2_AgBiBr_6_ double-perovskite photovoltaic cells has been limited by their low power conversion efficiency, with reported values being less than 6.37 on average. We conducted a comprehensive numerical study of the role of layer selection and parameter engineering in enhancing the photovoltaic response of hydrogenated Cs_2_AgBiBr_6_ in an inverted (p-i-n) structure in this work, with the aid of SCAPS-1D. Through the systematic optimization of the transport layers, electron transport properties, absorber properties, and hole transport layer properties, we observed that there were conditions where our devices could be greatly optimized to facilitate charge extraction and minimize recombination losses. It is worth noting that an ETL featuring SnO_2_ was replaced by ZnO, which resulted in significant gains in quantum efficiency and electrical functionality because the band alignment was better and the electron transport became more efficient. More elaboration of absorber thickness and defect density, including close regulation of HTL thickness and doping concentration, enabled further improvements of current density, fill factor and overall device efficiency. Simulated device with parameters optimized to include ZnO as the ETL and an Absorber Thickness of approximately 0.4 μm, a low density of the Defects to be the absorber (10^14^ cm^− 3^), a thin layer of Spiro-OMeTAD having optimal thickness of 0.01 μm, and increased doping of the HTL, a PCE of 25.92% was reached, which is a considerable improvement to lead-free double-perovskite photovoltaics. Those findings prove that the hydrogenated Cs_2_AgBiBr_6_ can acquire competitive activity in the case of a properly chosen material and optimized structure is considered. The results obtained offer many insights into the development of experiments and point to the significant potential of lead-free double-perovskite absorbers as sustainable options to be adopted in the future in photovoltaic systems.

## Data Availability

The data can be made available on reasonable request from the first authors.
